# Role of mitral valve repair in active infective endocarditis: long term results

**DOI:** 10.1186/s13019-017-0604-6

**Published:** 2017-05-18

**Authors:** Carlo Rostagno, Enrico Carone, Pier Luigi Stefàno

**Affiliations:** 1 Dipartimento Medicina sperimentale e clinica, Università di Firenze, Viale Morgagni 85, 50134 Florence, Italy; 2 Medicina Interna e Postchirurgica, AOU Careggi Firenze, Florence, Italy; 3 Cardiochirurgia, AOU Careggi Firenze, Florence, Italy

**Keywords:** Mitral valve surgery, Infective endocarditis, Heart failure, Survival

## Abstract

**Background:**

Although mitral valve repair is at present the technique of choice in mitral regurgitation (MR) due to degenerative valve disease, long term results in patients with active mitral infective endocarditis (IE) are still under evaluation.

**Methods:**

In the study were included 34 consecutive patients (22 males; mean age, 60 years; range 32–84 years) referred to our institution between January 1, 2005 to December 31, 2011 who were treated with valve repair for mitral valve (MV) active infective endocarditis. Eighteen patients underwent isolated MV repair. Aortic valve replacement and respectively repair were performed in 9 and 2 patients with concomitant aortic involvement. Blood cultures were positive in 30 (17 Staphylococcus, 13 Streptococcus, 1 g negative, 2 enterococcus).

**Results:**

Four patients died during hospital stay (11%) due to multi system organ failure as a consequence of severe septic shock (2 patients), cardiogenic shock (1 case) and respiratory failure (1 patient). At an average follow up of 48 months in patients discharged alive from hospital survival was 96.7% (29 out of 30). None developed more than mild- to moderate mitral valve regurgitation during follow-up and we found a significant improvement in functional capacity and left ventricular ejection fraction associated with a significant decrease of pulmonary artery pressure. The only recurrence of endocarditis occurred in a drug addict patient.

**Conclusions:**

Present investigation suggest that in patients with active mitral valve endocarditis MV repair, when technically feasible, is associated with a favorable clinical long term outcome. None of the patients alive at the end of follow-up developed severe mitral regurgitation. Moreover mortality and reinfection rate are uncommon and functional improvement.

## Background

Surgical treatment in mitral valve infective endocarditis (IE) is indicated in patients with severe mitral regurgitation, even in absence of congestive heart failure, with mitral annular abscess, large vegetation (>10 mm), uncontrolled sepsis and multiple emboli [1]. Mitral valve (MV) replacement has traditionally been considered the standard treatment for MV endocarditis unresponsive to antibiotic therapy, because of concerns about the durability of mitral valve repair, recurrence of IE, and mitral regurgitation [2–4]. Since the pioneer work by Dreyfus et al. [5] several authors suggested that in patients undergoing surgery for IE mitral valve repair may be safely performed and is often associated with a better outcome compared with mitral valve replacement [6–10]. A systematic review by Ferringa et al. [11] underlined that mitral valve repair was possible in approximately 39% of patients presenting with mitral valve endocarditis. Valve repair was associated with lower in-hospital and long term mortality [4, 6, 8]. Aim of present investigation was to evaluate at an average 4 years follow-up period the clinical and functional results of mitral valve repair in a group of consecutive patients with active mitral IE referred to our institution.

## Methods

In the study were included 34 consecutive patients referred to Heart Surgery Department of Azienda Ospedaliera Universitaria Careggi, Florence, Italy, a tertiary heart surgery referral center serving a population > 1.5 million, between January 1, 2005 and December 31, 2011 and treated with mitral valve repair for active infective endocarditis defined in agreement with the criteria of European Society of Cardiology [12]. Diagnosis of IE was made according to modified Duke University criteria [13] From the study were excluded patients undergoing mitral valve repair in healed endocarditis. Overall 34 patients, 22 males and 12 females, mean age 58 years (range 32–84 years) were enrolled in the investigation. The preoperative patient characteristics are reported in Table 1.

**Table 1:** Preoperative profile of the 34 patients with active infective endocarditis

Variable	
Age (years)	58 ± 17
Male/Female	22/12
LVEF %	57 ± 3
Systolic blood pressure mmHg	127 ± 13
Diastolic blood pressure mmHg	74 ± 7
Heart rate beats/min	81 ± 20
NYHA class	Patient number
I	3
II	9
III	17
IV	5
Aetiologic agent	
Staphylococcus	10
Streptococcus	17
Gram -	1
Enterococcus	2
Negative blood colture	4
Indication to surgery	
Peri valvular spreading of infection	10
Congestive heart failure	4
> 10 mm vegetations	16
Recurrent embolic event	4

At hospital admission 65% of patients was in New York Heart Association (NYHA) functional class III-IV, 26 and 9% respectively in class II and class I. Five patients had history of recent dental procedures, two patients were intravenous drug addicts. Causative microorganisms were isolated and identified in blood cultures in 30/34 patients. The aetiologic agents were Streptococcus species in 17 patients (50%) and Staphylococcus species in 10 (29,4%) patients. Antibiotic treatment was targeted according to results of antibiotic susceptibility testing. As suggested by the guidelines, large spectrum empiric antibiotic treatment was prescribed in patients with culture negative endocarditis [10, 11]. Indications to surgery has been: congestive heart failure with hemodynamic impairment in 4 patients, vegetation > 10 mm in diameter in 16, recurrent embolic event in 4 and persistent fever or perivalvular spreading of infection after at least 1 week of targeted antibiotic therapy in 10. All patients underwent surgery within 7 days after starting antibiotic treatment, unless impairment of clinical conditions suggested an earlier solution.

Informed consent was obtained from each patient and the study protocol conforms to the ethical guidelines of the 1975 Declaration of Helsinki as reflected in a priori approval by the institution’s human research committee.

Transthoracic and transesophageal (TEE) echocardiography were performed with a Sequoia Accuson instrument (Siemens Medical Solution, Mount View, California, USA). All examinations were performed by two senior echocardiographers adhering to American Society of Echocardiography guidelines [11]. Standard semi-quantitative method was used to assess the severity of regurgitation: absent, mild (<2+) or moderate-severe (3 + − 4+). Left ventricular ejection fraction (LVEF) was measured using area-lenght method. The examination was performed before and 2–3 days after surgery. Repair or replacement strategy were planned before surgery on the basis of anatomical characteristics and degree of tissue valve destruction and paravalvular extension of infection evaluated by trans esophageal echocardiography. After direct inspection in operating room only one patient scheduled for repair was shifted to valve replacement.

Surgical treatment:All procedures were performed through a median sternotomy and involved cardiopulmonary bypass with bicaval cannulation, Myocardial protection was identical for all patients and consisted of antegrade cold blood cardioplegia. The MV was exposed through a right-sided left atriotomy. The whole procedure was conducted under continuous TEE monitoring. The basic surgical principle was radical resection of all infected valvular and subvalvular tissue. 18 patients were treated with isolated MV repair. Associated procedure were performed in the remaining 16 patients including aortic valve replacement and repair, tricuspid valve annuloplasty, coronary artery bypass and a radiofrequency ablation procedure (Table 2). Triangular or quadrangular resection with sliding were used in 22 patients for anterior and posterior leaflet reconstruction. Glutaraldehyde-treated autologous pericardial patches were used in 6 patients and artificial chordal replacement was performed in 2 cases. The ring used for annuloplasty was autologous pericardium in 1 patient and prosthetic in 28 cases.

**Table 2:** Operative procedures

Procedures	n patient
Patch repair of AML	7
Patch repair of PML	1
Articial chordae	2
Annuloplasty:	
*Prosthetic ring*	28
*Autologous pericardium*	1
Quadrangular resection PML	16
Triangular resection PML	5
Triangular resection AML	1
Edge to edge	1
Associated operation	
Aortic valve replacement	9
Aortic valve repair	2
Coronary artery by pass	3
Maze operation	1
Tricuspid valve repair	1

*AML* anterior mitral leaflet, *PML* posterior mitral leafet

After discharge, all patients were admitted to a rehabilitation centre and thereafter followed by their general practitioner. Antibiotic treatment (according to antimicrobial susceptibility testing in patients with positive blood cultures and empirical in patients with negative blood cultures) was prosecuted for at least 6 weeks after surgery. Clinical examination were scheduled (3, 12, 24, 32 and 48 months). The mean follow-up period after discharge has been 4 years.

### Statistical analysis

The quantitative variables are reported as means and standard deviations. In case of not continuous parameters the frequency of distribution has been reported. The statistical analysis of clinical data was carried out by the Student’s *t* test for continuous data, while for not continuous variables *χ* ^2^ test was used. The analysis of survival was made using the Kaplan- Meier method. A probability value of < 0.05 was considered statistically significant.

## Results

Four of 34 patients included in the study died during hospital stay (11%). All deaths were due to multisystem organ failure as consequence of severe septic shock (2 patients), cardiogenic shock (1 case) and respiratory failure (1 patient). In 2 patients, the microorganisms responsible for endocarditis was a methicillin-resistant staphylococcus aureus, in one case a Streptoccoccus parasanguinis and in the last patient blood culture was negative.

**Fig. 1:**
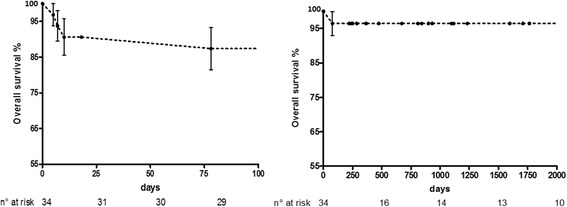
Left survival curve the first 100 days after surgery. Left – long term survival curve in patients discharged alive from the hospital

In patients discharged alive from hospital survival was 96.7% (29 out of 30) at an average follow up of 48 months (Fig. 1). One patient underwent double valve surgery (mitral valve repair and Bentall’s intervention) died 3 months postoperatively of sudden death. Endocarditis recurred only in 1 patient (intravenous drug user) 11 months postoperatively; he needed reintervention.

Surgical treatment led to a significant functional improvement. At the end of the follow-up 90% (27 out of 30) of the patients discharged alive from hospital were in NYHA class I, II and all the patients were in sinus rhythm. Transthoracic echocardiography showed a significant decrease of left ventricular end diastolic diameter, an improvement of left ventricular ejection fraction and decrease of pulmonary artery pressure. At last examination none developed severe mitral regurgitation, 20 patients had mild (≤2+) and 9 had no or trivial mitral valve regurgitation. Pulmonary systolic pressures were significantly diminished at the time of follow-up. Pre- and postoperative echocardiographic data are summarized in Table 3.

**Table 3:** Preoperative and follow-up echocardiographic and functional parameters

Parameter	Preoperative	Follow up	
LV Ejection fraction (%)			
> 50%	26	29	
35–50%	4	0	*χ* ^2^ 5.472
< 35%	4	0	*p* 0.065
Pulmonary systolic pressure (mmHg)			
> 50 mmHg	6	0	
30–50 mmHg	19	14	*χ*2 7.264
< 30 mmHg	9	15	*p* 0.0265
LV end diastolic diameter (mm)			
< 55 mm	17	24	
55–60 mm	3	5	*χ*2 16.92
> 60 mm	14	0	*p* 0.0002
Mitral regurgitation			
0	0	9	
< 2+	4	20	*χ*2 47.57
3+ 4+	30	0	*p* < 0.0001
NYHA functional class			
I	3	17	
II	9	10	*χ*2 25.69
III	17	2	*p* < 0.0001
IV	5	0	

## Discussion

In agreement with more recent data suggesting a decreased risk of mortality, stroke, recurrent endocarditis and reoperation after valve repair [6–11], in our institution we had a progressive trend towards conservative treatment also in patients with active endocarditis. At present less than 40% of mitral valve endocarditis undergo mitral valve replacement.

Resection technique should allow excision of enough leaflet tissue to ensure eradication of the infectious process. Anterior leaflet lesions, commissural lesions, extensive leaflet destruction and paravalvular abscess may require different and often complex techniques for MV repair [14]. Annuloplasty using a prosthetic ring provides coaptation of leaflets, limits tension in the suture, prevents further dilation of the annulus allowing a stable late surgical result [2, 6, 8]. Autologous pericardium is an alternative material for annuloplasty in patients with active IE. We used autologous pericardium for annuloplasty in one patient. MV repair, maintaining the annular-chordal-papillary muscle continuity, may allow to obtain more frequently the recovery of left ventricle geometry in comparison to mitral valve replacement, moreover it decreases the risk related to prolonged anticoagulation [15]. Several studies have shown that valve repair in IE is associated with lower in-hospital and long term mortality. Moreover a decreased risk of stroke, recurrent endocarditis and reoperation has been reported [8, 11].

Omoto et al. [16] performed valve repair in 68% (15/22) of patients with mitral valve endocarditis. Seven out of 15 cases required a complex repair. 3/15 (20%) patients required reintervention due to dehiscence of the pericardial patch. Ruttman et al. [17] studied 68 consecutive patients needing surgery for mitral endocarditis. Thirty-four (50%) patients had valve repair, and 34 (50%) patients had valve replacement. Hospital mortality was 11.8% (8 patients). No significant differences were found in all baseline parameters, with the exception of a higher incidence of previous septic embolism and sepsis in the repair group. At 1 year actuarial event-free survival was 88.2% in the repair group compared with 67.7% in the replacement group. In the study by Kanemitsu et al. [18] 43 patients who underwent primary MV repair for IE at a single center between 1991 and 2009 were retrospectively examined. Survival was 92.6 ± 4.1% at 5 years. The rates of freedom from MV reoperation was 90.5 ± 4.5% and for freedom from moderate or severe MR was 95.0 ± 3.5%. Recurrence of endocarditis was observed in 2 patients (4.7%). Most (86%) of the survivors were in NYHA class I.

MV repair was possible in 60% of patients with active mitral IE referred to our institution. Despite antibiotic treatment and early surgical treatment post-operative mortality in patients with active-phase infective endocarditis remain high. In present study in hospital mortality was 11%, similar to that reported in previous studies (8–12%). Long term survival of discharged patients, 96.7%, is similar to that reported by Kanemitsu [18] and higher than in other studies [11, 17]. All patients showed a significant improvement in functional capacity. 93.2% were in NYHA functional class I-II at follow-up. In our investigation none patients developed more than mild to moderate mitral regurgitation, while at least a 5% of severe regurgitation was found at follow-up in other studies [11, 16, 19]. As shown by Ruttmann et al. [17] mitral valve repair is associated with a lower rate of reinfection in comparison to mitral valve replacement; 4% vs 17%. A high risk condition (drug addiction) was present in the only patient with recurrent IE observed in our study. The use of prosthetic material in MV repair in active IE is still controversial since the use of a prosthetic annuloplasty ring may be associated with a higher risk of endocarditis recurrence. A prosthetic ring was used in 28/29 patients and we did not find any device related infection.

Streptococci were the more frequent etiologic agents (50%) in our study, in agreement with previous investigations [16, 18]. It is conceivable that a more limited damage to valve structure related to the lower virulence of streptococcal infection allowed more frequently to perform MV repair.

## Limitation to the study

The absence of a control group of patients undergoing mitral valve replacement for active infective endocarditis does not allow a direct comparison of the two surgical techniques. It is likely that the study may suffer the bias that patients with fewer comorbidities and less aggressive infection were selected for repair. The superiority demonstrated by mitral valve repair over valve replacement in the only study that directly compared these 2 surgical techniques [17] makes ethically difficult to suggest a randomized study. Although characteristics of the groups in the randomized study did not significantly differ as the authors state “it is very difficult to be sure that the characteristics of the patients and, especially, of the valves are identical. Also, we cannot neglect effects caused by the surgeon’s experience.”

## Conclusion

MV repair is an attractive surgical option in patients with active IE. Expertise of surgical team and favorable anatomical conditions allowed to preserve mitral valve in 60% of patients referred to our institution. Results from present study suggest that mitral valve repair in active-phase infective endocarditis is associated with a good long-term outcome (high long-term survival, significantly recovery of functional capacity, low reinfection rate). Moreover at the end of follow-up no patients showed severe valve regurgitation. In conclusion, in our opinion, when technically feasible mitral valve repair should be preferred to mitral valve replacement in patients with active mitral IE.

## Abbreviations

IE: Infective endocarditis
LVEF: Left ventricular ejection fraction
MR: Mitral regurgutation
MV: Mitral valve
NYHA: New York Heart Association
TEE: Transesophageal echocardiograèhy
